# Investigation of Water Dynamics and the Effect of Evapotranspiration on Grain Yield of Rainfed Wheat and Barley under a Mediterranean Environment: A Modelling Approach

**DOI:** 10.1371/journal.pone.0131360

**Published:** 2015-06-22

**Authors:** Kefeng Zhang, Angela D. Bosch-Serra, Jaume Boixadera, Andrew J. Thompson

**Affiliations:** 1 Ningbo Institute of Technology, Zhejiang University, Ningbo, China; 2 Department of Environmental and Soil Sciences, University of Lleida, Lleida, Spain; 3 Department of Agriculture, Livestock, Fisheries, Food and Natural Environment, Generalitat de Catalunya, Lleida, Spain; 4 Cranfield Soil and AgriFood Institute, School of Applied Sciences, Cranfield University, Bedfordshire, United Kingdom; DOE Pacific Northwest National Laboratory, UNITED STATES

## Abstract

Agro-hydrological models have increasingly become useful and powerful tools in optimizing water and fertilizer application, and in studying the environmental consequences. Accurate prediction of water dynamics in such models is essential for models to produce reasonable results. In this study, detailed simulations were performed for water dynamics of rainfed winter wheat and barley grown under a Mediterranean climate over a 10-year period. The model employed (Yang et al., 2009. J. Hydrol., 370, 177-190) uses easily available agronomic data, and takes into consideration of all key soil and plant processes in controlling water dynamics in the soil-crop system, including the dynamics of root growth. The water requirement for crop growth was calculated according to the FAO56, and the soil hydraulic properties were estimated using peto-transfer functions (PTFs) based on soil physical properties and soil organic matter content. Results show that the simulated values of soil water content at the depths of 15, 45 and 75 cm agreed with the measurements well with the root of the mean squared errors of 0.027 cm^3^ cm^-3^ and the model agreement index of 0.875. The simulated seasonal evapotranspiration (ET) ranged from 208 to 388 mm, and grain yield was found to correlate with the simulated seasonal ET in a linear manner within the studied ET range. The simulated rates of grain yield increase were 17.3 and 23.7 kg ha^-l^ for every mm of water evapotranspired for wheat and barley, respectively. The good agreement of soil water content between measurement and simulation and the simulated relationships between grain yield and seasonal ET supported by the data in the literature indicates that the model performed well in modelling water dynamics for the studied soil-crop system, and therefore has the potential to be applied reliably and widely in precision agriculture. Finally, a two-staged approach using inverse modelling techniques to further improve model performance was discussed.

## Introduction

With the advances in plant and soil sciences and in computing power, developing physically-based agro-hydrological models have become possible and numerous models of this kind have been developed [1–2]. These models have proven useful and powerful in precision agriculture, and in studying the diverse impact on the environment induced by excessive application of resources. As the core of agro-hydrological models, modelling water dynamics in the soil-crop system is critically important and has a profound effect on the accuracy of model outcomes.

Great efforts have been directed to developing models for water dynamics in the soil-crop system and numerous models have been reported in a large body of literature [1, 3–5]. In modelling soil-cereal systems, some specially designed simulation models for water and nitrogen dynamics have been proposed, including AFRCWHEAT2 [6–7], CERES-Wheat [8–9], NWHEAT [10], SIRIUS [11], SOILN-Wheat [12–13], AGROSIM [14] and HERMES [15]. While these models, if validated properly, have proven useful [16–18], they generally use dozens of parameters describing crop physiological processes. The models require calibration under different climates and the parameters are often difficult to obtain.

In contrast with those models, a simpler model which uses readily available agronomic data has been devised and tested against data from winter wheat grown in different soils and under a semi-humid climate [19]. The model defines the crop growth stages and employs the dual crop efficient approach according to the FAO56 [20] for estimating water requirement for crop growth. The simulation of soil water movement is performed by solving the Richards’ equation with a simple numerical scheme named the Integrated Richards Equation (IRE) method proposed by Boone and Wetzel [21] and Lee and Abriola [22]. Although the model has shown promising results in reproducing soil water data from the winter wheat experiments mentioned the above [19], the potential of the model has not been fully explored as it has not been tested in different climates over different ranges of water supply.

Investigations have been carried out on the effect of seasonal evapotranspiration (ET) on grain yield for wheat and barley experimentally and using modelling approaches [23–29]. It is well documented that the total dry weight yield and/or grain yield of wheat and barley increases with increasing seasonal ET if water is limiting [23, 24–30]. Many studies reported that grain yield could be related with seasonal ET linearly [23, 24, 29–30], as found in other crops [31–32]. Although it appears that the linear relationship between wheat grain yield and seasonal ET is well established, it is often based on the data collected from the crops grown over short-term experiments with a narrow range of ET. Further, the estimation of seasonal ET is simply performed using soil water content in the profile measured at sowing and harvest and seasonal rainfall, ignoring soil water movement between the root zone and the deep soil.

The primary purposes of this study are therefore: 1) to further test the capability of the model [17] to simulate water dynamics in the soil-wheat/barley system under a Mediterranean climate; and 2) to establish the relation between wheat/barley grain yield with seasonal ET using data from a long-term study.

## Materials and methods

### Experiments

A long-term experimental study on wheat and barley over 10 years (2000–2010) was conducted at Lleida University in Spain. The experiments were carried out on a non-protected field and did not involve endangered or protected species. The primary purpose of the study was to investigate the effects of water, mineral and organic nitrogen (N) fertiliser on winter wheat and barley production and the environmental impacts. The experimental site is located in Oliola, Lleida, Catalonia, Spain (41°52’34” N, 0°19’17” E), within a region mainly growing cereal crops. The site (443 m above the sea level) is open and flat. The region is characterized as a semi-arid Mediterranean climate [33] with a mean annual temperature of 12.6°C and a mean annual precipitation ranging from 292 and 593 mm (2000–2010 period). The soil, having a thickness of more than 1 m throughout the field, is classified as a Typic Xerofluvent [34] with the top soil of about 30 cm (see Table 1 for the measured soil physical properties). The groundwater table is well below a depth of 2 m.

**Table 1:** Soil particle distribution and organic matter content in the profile.

-	Bulk density	Clay (<0.002mm)	Silt (0.002 ~ 0.05mm)	Sand (0.05 ~ 2mm)	Organic matter
-	g cm^-3^	%	%	%	%
0–30cm	1.65	20.3	52.1	27.6	1.7
30cm ─	1.57	16.4	49.0	34.7	0.7

The size of the experimental field was about 1.5 ha. The experiments, starting from 2000, had been carried out for 10 years on winter wheat and barley, except for the year 2007–2008 when the soil was set fallow. Soil water monitoring started from November 2005 and soil water content values were recorded at the depths of 15, 45 and 75 cm using the ECH_2_O sensors with an error of ±3% and Em50G data loggers (Decagon Devices). Due to some technical problems and faults with the sensors during the experiments, the sensor measurements were not complete over the entire monitoring period, but give sufficient data for model validation.

Five treatments using different amounts of mineral N fertilizer as NH_4_NO_3_, i.e 0, 30, 60, 90, and 150 kg N ha^-1^, were designed in the experiments to study the response of crop yield to N fertiliser. The results reported in this study are only part of the systematic experimentation. To exclude the diverse effect of insufficient and excessive N fertiliser application on crop growth, the crop grain yield was obtained based on the measurements from the treatments where sub-optimal amounts of N fertilizer were applied. Total above-ground biomass was measured from 4 randomly selected plots each having an area of 0.25 m^2^ at maturity. Biomass was separated into stems and leaves, spikes and grains and was oven-dried at 65–70°C for 48 h before being weighed. Table 2 shows the crop rotations during the experimental period together with the sowing and harvest dates and the grain yield of each crop.

**Table 2:** Summary of experiments from 2000–2010.

Exp year	2000–2001	2001–2002	2002–2003	2003–2004	2004–2005
Crop	Barley	Barley	Wheat	Barley	Barley
Sowing date	05/11/00	08/11/01	31/10/02	30/10/03	16/11/04
Harvest date	18/06/01	26/06/02	26/06/03	02/07/04	01/07/05
Grain yield (t ha^-1^)	3.66	3.79	2.40	3.62	1.25
Exp year	2005–2006	2006–2007	2008–2009	2009–2010	-
Crop	Wheat	Barley	Wheat	Barley	-
Sowing date	04/11/05	04/11/06	10/11/08	03/11/09	-
Harvest date	27/06/06	26/06/07	04/07/09	30/06/10	-
Grain yield (t ha^-1^)	2.85	2.85	5.60	6.54	-

**Fig 1:**
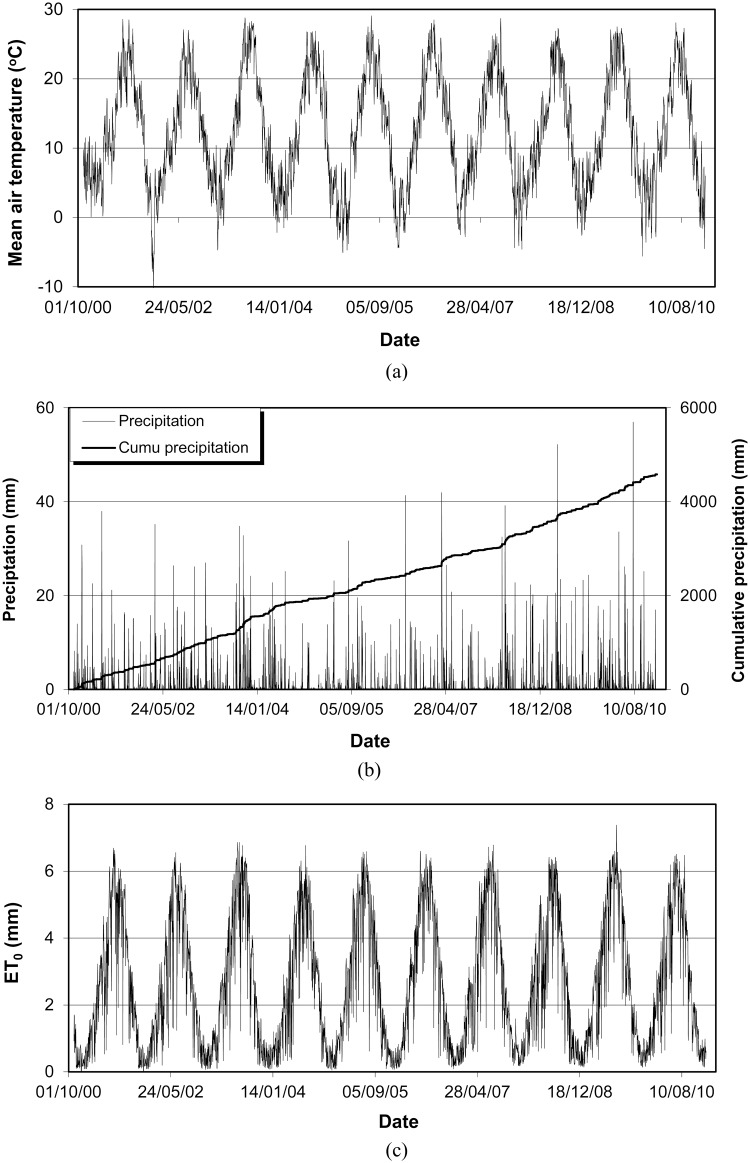
Measured daily mean air temperature (a), daily and cumulative precipitation (b) and calculated daily reference evapotranspiration (ET_o_) (c) during 2000–2010.

Weather data was collected from a nearby weather station belonging to the Catalonian Agrometeorological station network, which is 100 m away from the experimental field. Fig 1 and S1 Table. show the measured daily mean air temperature, precipitation and the calculated reference evapotranspiration using Penman—Monteith method [20] over the experimental period from 2000 to 2010.

### The model

#### Brief description of the model

The model used in this study, the source code for which is available at request from the authors, was proposed by Yang et al. [19] and Zhang et al. [35]. The model is mechanistic and accounts for all key processes governing water dynamics in the soil-crop system. It simulates soil water movement by solving the Richards’ equation using a simple and sufficiently accurate solution. The model has been validated in simulating water dynamics for winter wheat grown in different soils [19], and cabbage grown in a sandy loam soil under semi-humid climates [35]. It uses two time steps for various soil and plant processes in the model. On a daily basis, the model calculates:

- Rooting depth and relative root length distribution: the rooting depth is calculated based on the cumulative mean day air temperature according to Pedersen et al. [36]. Root growth starts when the cumulated effective day air temperature exceeds the threshold value. The effective day temperature is calculated as the difference between mean air temperature and the base temperature below which the plants do not grow. The rooting depth increase is the product of the effective day temperature and specific root growth rate [35]. The relative root length density is assumed to decline logarithmically from the soil surface downwards as shown in Gerwitz and Page [37] and Pedersen et al. [36].
- Potential evaporation and crop transpiration: the potential reference evapotranspiration (ET_o_) is first calculated using the FAO Penman-Monteith equation [20] based on the climatic variables of solar radiation, air temperature, relative humidity, wind speed and the latitude and altitude of the location. Potential evaporation and crop transpiration are then calculated according to the crop growth stages using the FAO dual crop coefficient approach [20].

The model implements the following algorithms for calculating soil evaporation, crop transpiration and soil water movement using a small time step (0.001d) [19] within the day. The biggest advantage of using this approach is that this allows the simultaneous processes of root water uptake and soil water movement to be de-coupled and a simple algorithm named Integrated Richards Equation (IRE) [22] to be implemented for soil water movement.

- It computes actual evaporation in the top soil layer and root water uptake in the root occupied layers according to soil water availability [38].
- It applies the IRE algorithm to re-distribute soil water in the simulated domain. Water movement in the profile can be downwards as well as upwards depending on the soil water pressure head in the adjacent layers. The thickness of each soil layer is 5 cm which is considered appropriate and commonly used in agro-hydrological models [39–41] to describe processes such as root length distribution in the soil-crop system.

**Fig 2:**
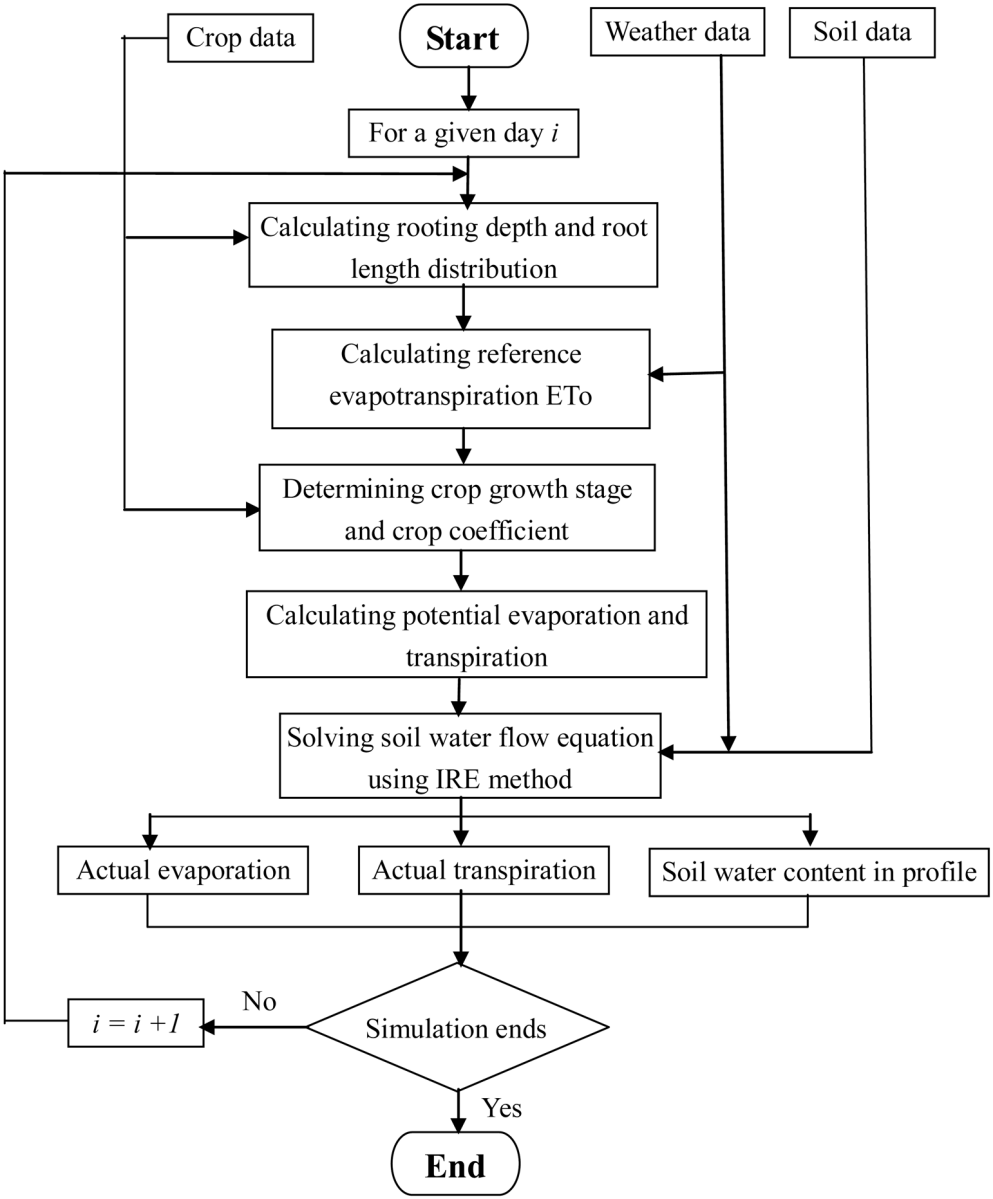
Schematic diagram of calculation procedures in the model used for soil-crop water dynamics in this study.

Fig 2 shows the flow chart of the model used in the study. The detailed description of the model and associated equations are given in Yang et al. [19] and Zhang et al. [35].

#### Model parameters and the values used in the simulations

The input parameters to the model are relatively simple and easy to collect. They include:

- Site geographical properties: latitude and altitude.
- Simulation period: the dates when the simulation starts and ceases.
- Weather data: daily mean air temperature, relative humidity, precipitation, solar radiation, wind speed.
- Soil hydraulic properties: van Genuchten [42] soil hydraulic properties defining the relationships of soil water content and hydraulic conductivity with water pressure head for the soils in the computed domain.
- Crop data: dates for sowing/transplanting and harvest.
- Initial conditions: soil water content/potential distribution in the profile.

Soil hydraulic properties can be estimated based on soil physical properties with reasonable accuracy, and various models have been proposed on this purpose [43–44]. The van Genuchten [42] soil hydraulic properties used in this study were estimated by the peto-transfer functions (PTFs) proposed by Wösten et al. [45]. The PTFs use the information of soil particle distribution, bulk density and organic matter content. They are derived based on a study of extensive EU soil samples, and are considered particularly appropriate for the soils used in the study. The estimated soil hydraulic properties for the topsoil and subsoil are listed in Table 3. Other parameter values used in the simulations for both the barley and wheat experiments and how they were obtained are given in Table 4. The calculated soil domain is down to 200 cm, and the condition at the lower boundary is set as free drainage.

**Table 3:** Van Genuchten soil hydraulic properties.

-	*θ*_*s*_ ^a^	*θ*_*r*_ ^a^	α^b^	*n* ^b^	*K*_*s*_ ^c^
-	(cm^3^ cm^-3^)	(cm^3^ cm^-3^)	(-)	(-)	(cm d^-1^)
0–30cm	0.36	0.025	0.01527	1.1744	7.6
30cm ─	0.38	0.025	0.01985	1.2661	12.3

^a^ *θ*_*s*_, *θ*_*r*_: the saturated and residual soil water contents, respectively.

^b^ *α*, *n*: the shape parameters of the retention and conductivity functions, respectively.

^c^ *K*_*s*_: the saturated hydraulic conductivity.

**Table 4:** Model parameter values used in the simulations.

Symbol	Unit	Value	Notation	Reference
*T*_*thre*_	d °C	100.0	threshold of cumulated effective day temperature for crop to start growth	[46]
*α*_*z*_	(-)	3.0	shape parameter controlling root distribution in soil profile	[19]
*k*_*rz*_	cm d^-1^ °C^-1^	0.07	root growth rate per effective day temperature	[19]
*T*_*base*_	°C	7.0	base air temperature for crop growth	[39]
*P*	(-)	0.55	soil water depletion fraction for no stress	[20]
*L*_*ini*_	d	30	duration in days for different crop growth stages (initial, development, mid-season and late season)	[20]
*L*_*dev*_	d	140		
*L*_*mid*_	d	40		
*L*_*lat*_	d	30		
*K*_*cb ini*_	(-)	0.15	basal crop coefficient corresponding to the initial, mid-season and late growth stages	[20]
*K*_*cb mid*_	(-)	1.10		
*K*_*cb end*_	(-)	0.15		

#### Model evaluation criteria

Accuracy of the model predictions of soil water content against the measurements was evaluated based on statistical analyses. The statistical indices used for the model assessment include: the root of the mean squared errors (*RMSE*), the model agreement index (*AI*) [47], the mean error (*ME*), the correlation coefficient (*r* ^*2*^) and the slope and intercept of the fitted line between simulated and measured values. The software used for statistical analyses was developed by the authors.

$$RMSE=\sqrt{\sum_{i=1}^{n}{\left(o_{i}-s_{i}\right)}^{2}/n}$$ (1)

$$AI=1-\sum_{i=1}^{n}{\left(o_{i}-s_{i}\right)}^{2}/\sum_{i=1}^{n}{\left(\left|s_{i}-o'\right|+\left|o_{i}-o'\right|\right)}^{2}$$ (2)

$$ME=\sum_{i=1}^{n}\left(s_{i}-o_{i}\right)/n$$ (3)

where *n* is the number of samples, *o*_*i*_ and *s*_*i*_ are measured (observed) and simulated values, and *o’* is the average of the measured values.

## Results and Discussion

### Assessment of overall model performance

**Fig 3:**
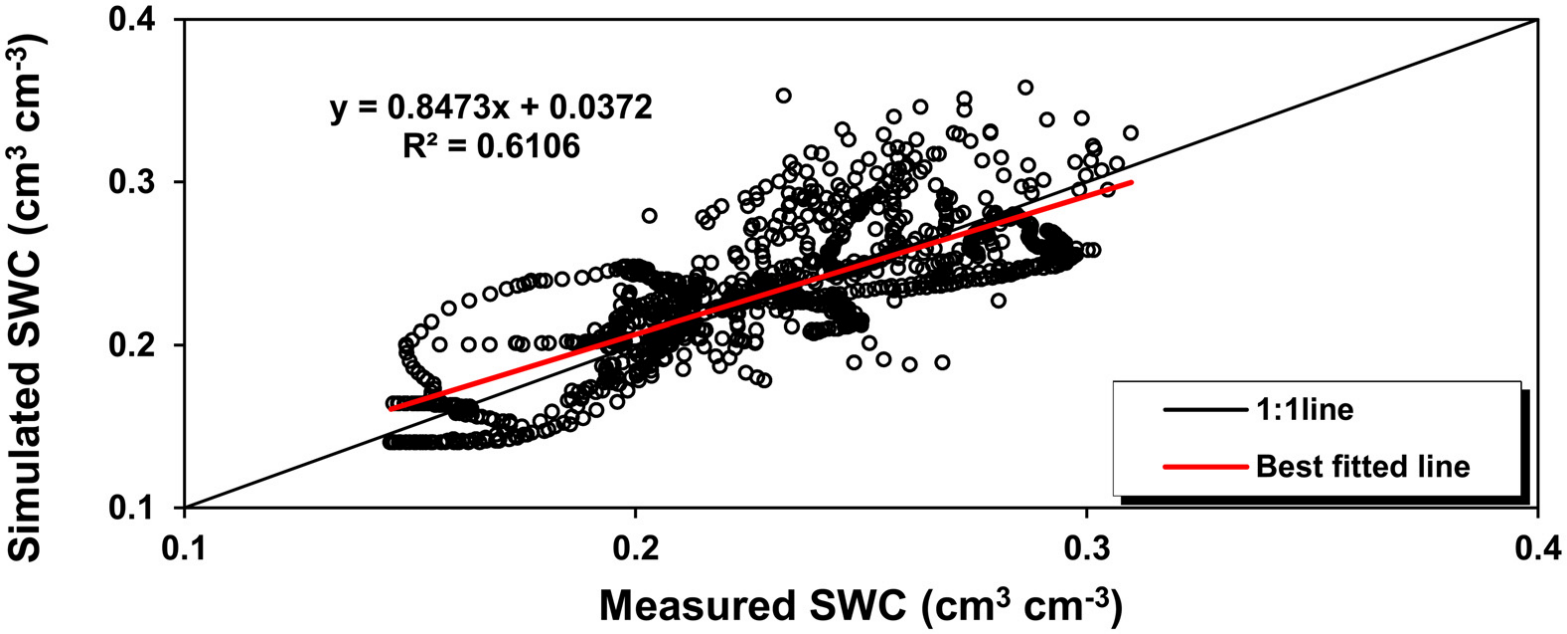
Overall comparison of soil water content (SWC) between simulation and measurement.

The model was run without making any adjustment of parameter values that might improve the degree of agreement between measurement and simulation. Fig 3 shows the overall comparison of simulated and measured values of soil water content at various depths for the experiments in 2006–2010. It can be observed that not only are the simulated values correlated with the measured values fairly well (*r* ^*2*^ = 0.611, *n* = 1169), but also the best fitted line gives the gradient close to 1 (0.847) and a very small intercept of 0.037 cm^3^ cm^-3^, indicating that the model is able to reproduce measurements reasonably well. The overall good performance of the model is also confirmed by the calculated statistical indices (Table 5). The *RMSE* value, a representative deviation of the simulated values from the measurements, is only 0.027 cm^3^ cm^-3^, while the model agreement index *AI* gives a high value of 0.875, greater than 0.8 to be considered as model good performance [47]. The model overestimates the values of soil water content, but only by a negligible margin as the mean error *ME* between simulation and measurement is 0.004 cm^3^ cm^-3^. It is, therefore, reasonable to conclude that the model performs well in predicting soil water dynamics during crop growth, and thus is sufficiently reliable to be applied in modelling the water cycle for the entire experimental period.

**Table 5:** Statistical indices of soil water content between measurement and simulation.

*n*	*RMSE*	*ME*	*AI*	Slope	Intercept	*r* ^*2*^
(-)	(cm^3^ cm^-3^)	(cm^3^ cm^-3^)	(-)	(-)	(cm^3^ cm^-3^)	(-)
1169	0.027	0.002	0.876	0.847	0.042	0.611

### Comparison of measured and simulated soil water content

**Fig 4:**
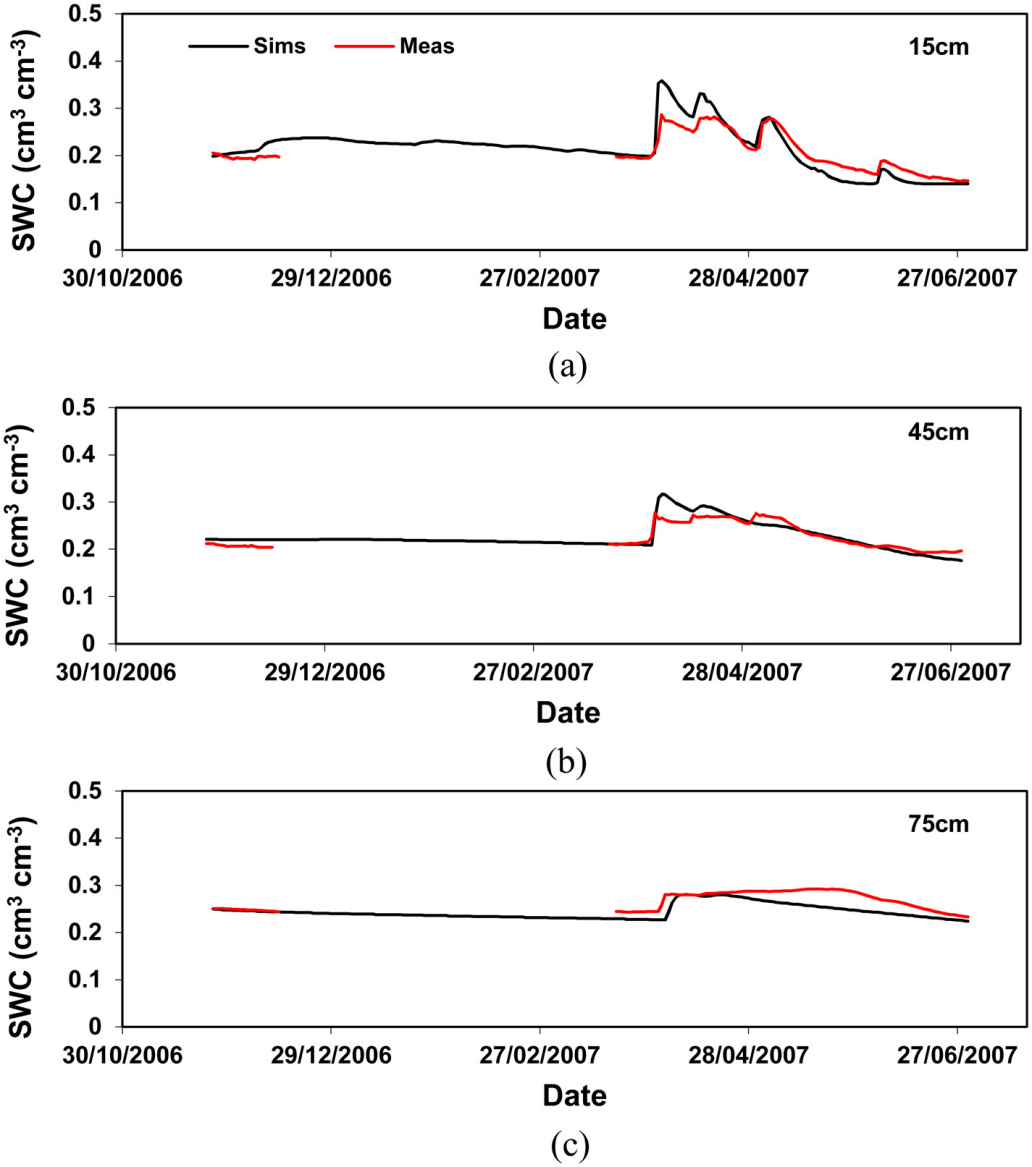
Comparison of soil water content at the depths of 15 cm (a), 45 cm (b) and 75 cm (c) in the 2006–2007 experiment.

**Fig 5:**
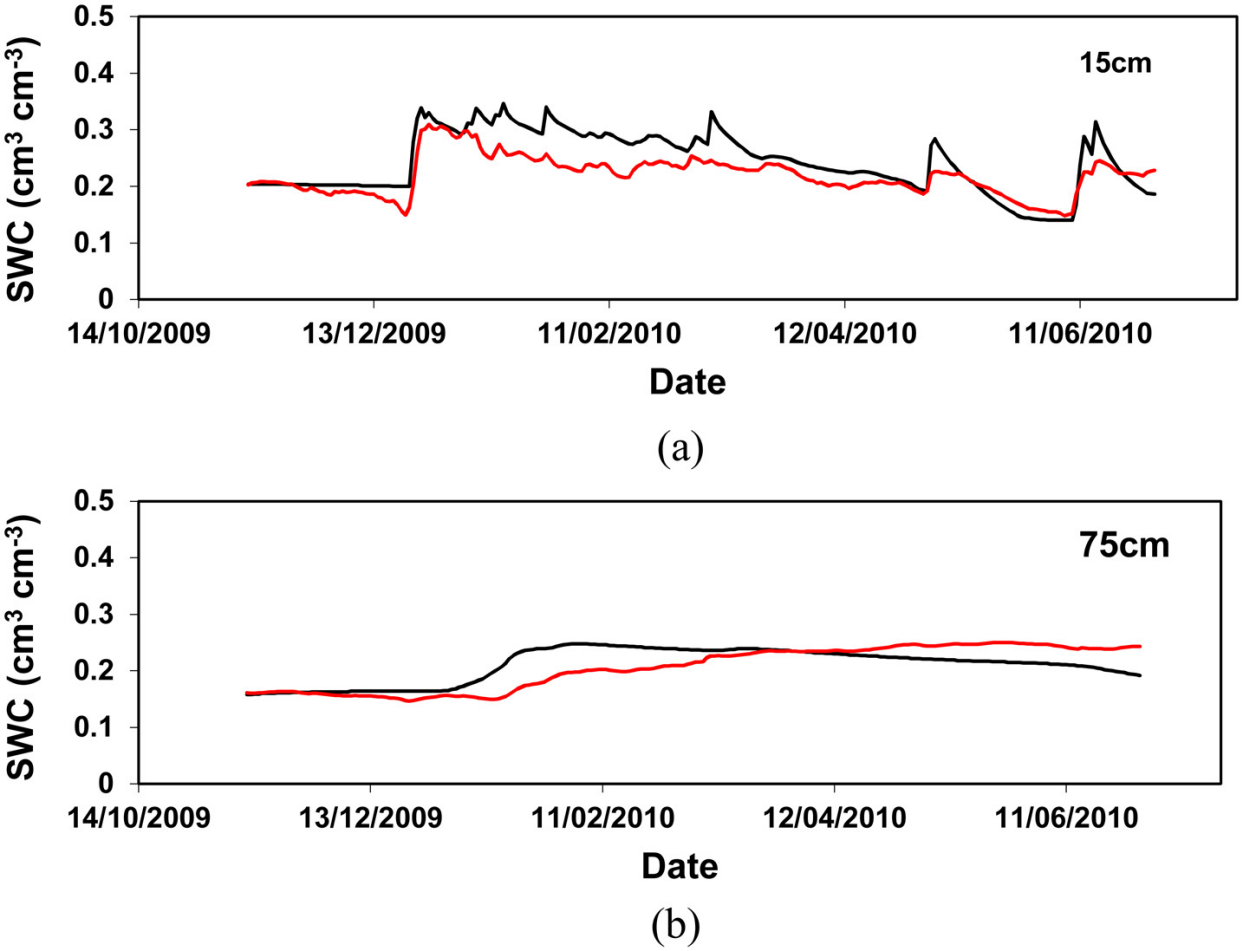
Comparison of soil water content at the depths of 15 cm (a) and 75 cm (b) in the 2009–2010 experiment.

Detailed comparisons of the soil water content at various depths between measurement (S2 and S3 Tables.) and simulation were carried out for the experiments in 2006–2010, and are shown in Figs 4 and 5 as examples. Generally, it is clear that the model reproduced the measurements of soil water content well. The big increases in soil water content that occurred between 1 to 5 April 2007 (Fig 4) and between 21 to 24 December 2009 at 15 cm depth (Fig 5), coincided with a wet spell of weather, were correctly simulated. The change in soil water content appears to be less drastic in the subsoil than the topsoil, suggesting soil water was affected by rainfall more markedly in the topsoil as expected. While the overall performance of the model in reproducing the measurements is reasonably good, discrepancies of varying degrees also exist between measurement and simulation. The biggest discrepancies occur at the 75 cm depth in the experiment 2009–2010. After a wet spell from 21 to 24 December (52.2 mm rainfall in total), the model simulated a gradual and steady increase of water content at the 75 cm depth from 06 January 2010. However, the measurements show the increase in soil water content started later (14 Jan.) and at a less rapid pace. This might be attributed to the soil hydraulic properties estimated using the PTFs in this study. Although the PTFs were derived based on extensive EU soil samples [45], accurate determination of soil hydraulic properties still remains a big challenge. In fact, this is not a problem solely from the PFTs approach because the same problem exists for other ways of determining soil hydraulic properties such as the direct measurements of soil cores. The difficulties in making satisfactory estimates of soil hydraulic properties at a field scale have become a major obstacle to the taking-up of physically-based agro-hydrological models for practical uses [1]. Fortunately, new ways of estimating soil water properties using inverse modelling techniques have been proposed and received enormous efforts [48–52]. Such techniques have proven promising to estimate the parameters required by mechanistic agro-hydrological models [1].

### Potential and simulated evapotranspiration

**Fig 6:**
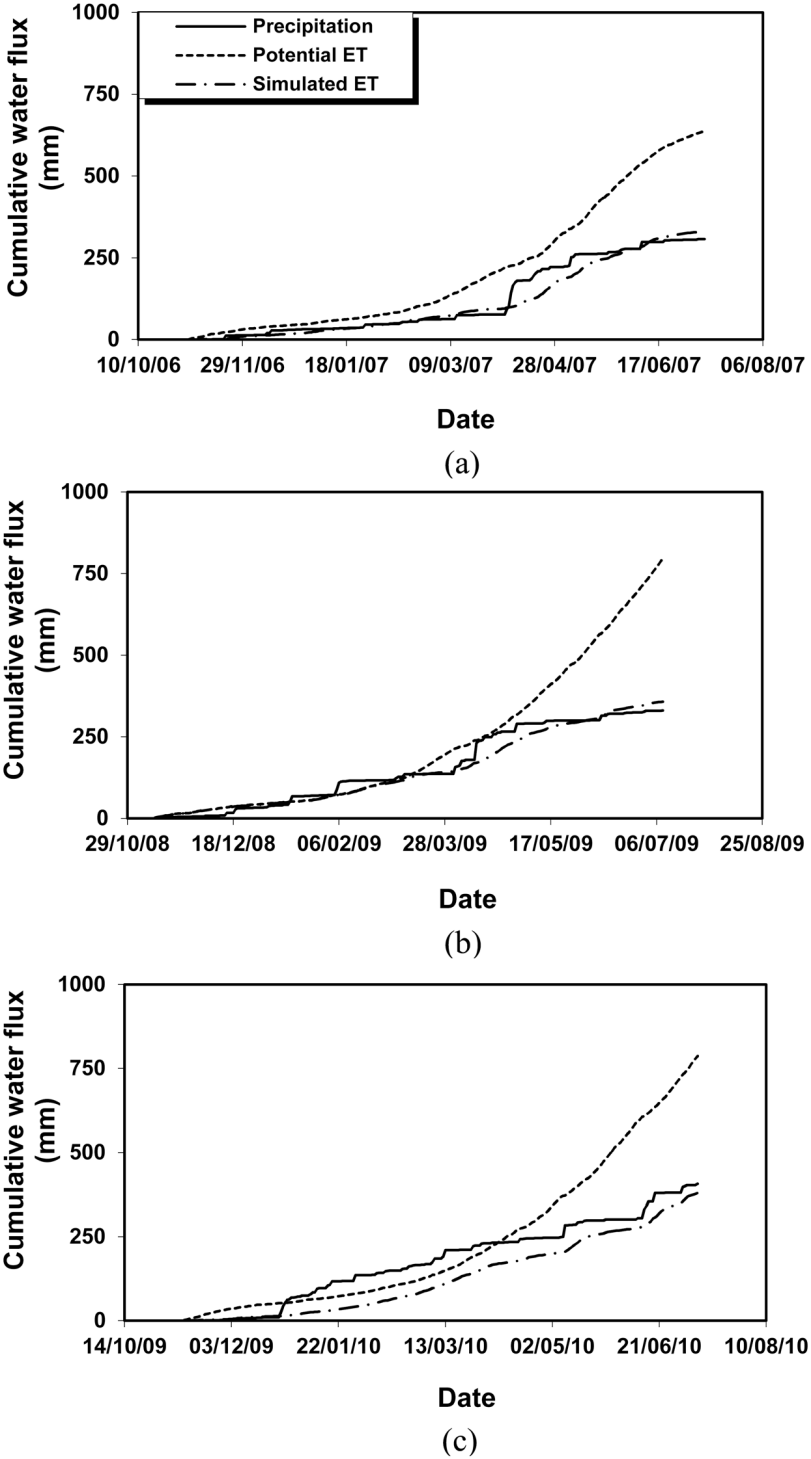
Cumulative precipitation, potential and simulated ET in the experiments 2006–2007 (a), 2008–2009 (b) and 2009–2010 (c).

Fig 6 shows the cumulative daily potential ET and the simulated ET in the growing seasons (the period between sowing and harvest), together with the precipitation for the experiments in 2006–2010. Generally it is the case that seasonal potential ET exceeded the simulated ET by far, suggesting that the crops suffered from water stress rather severely. This is due to the shortage of precipitation to meet the water demand for crops to achieve the maximum growth. Further, it is revealed that the simulated seasonal ET was approximately equal to the total precipitation in the same period. For example, for barley harvested on 26 June 2007, only 52% of seasonal potential ET of 639 mm, i.e 333 mm, was achieved, close to 307 mm provided by precipitation in the same period (Fig 6a).

**Fig 7:**
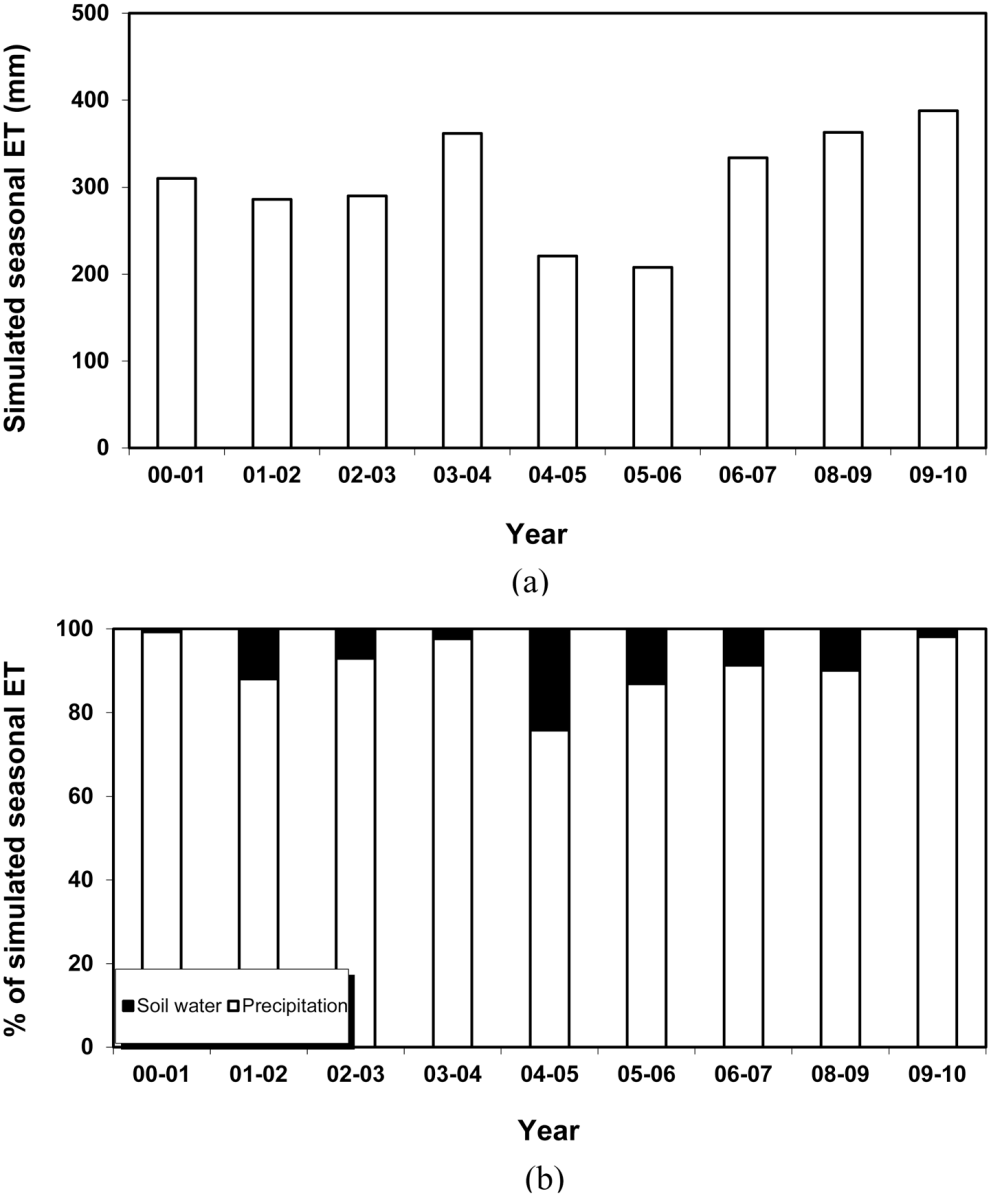
Simulated seasonal ET (a) and the contributions of precipitation and soil water to the simulated seasonal ET (b) from 2000–2010.

The simulations of seasonal ET over the entire experimental period and the contributions to the simulated ET by precipitation and soil water initially contained in the profile were performed (Fig 7). The simulated seasonal ET varied from 208 mm in the experiment 2005–2006 (precipitation of 180 mm) to 388 mm in the experiment 2009–2010 (precipitation of 380 mm) (Fig 7a), and the proportion made by initial soil water to the simulated seasonal ET was in the range of 24.2% from the dry growing season in 2004–2005 to 0.7% from the relatively wet growing season in 2000–2001 (Fig 7b), with the mean contribution to the simulated seasonal ET of 8.8% over all seasons. This indicates that the simulated seasonal ET is highly dependent on the precipitation in the growing season, and was mainly met by the precipitation rather than the soil water initially contained in the profile. This is in agreement with previous studies on cereal grown in the similar climate [53–54].

### Relationships between crop grain yield and simulated seasonal ET

**Fig 8:**
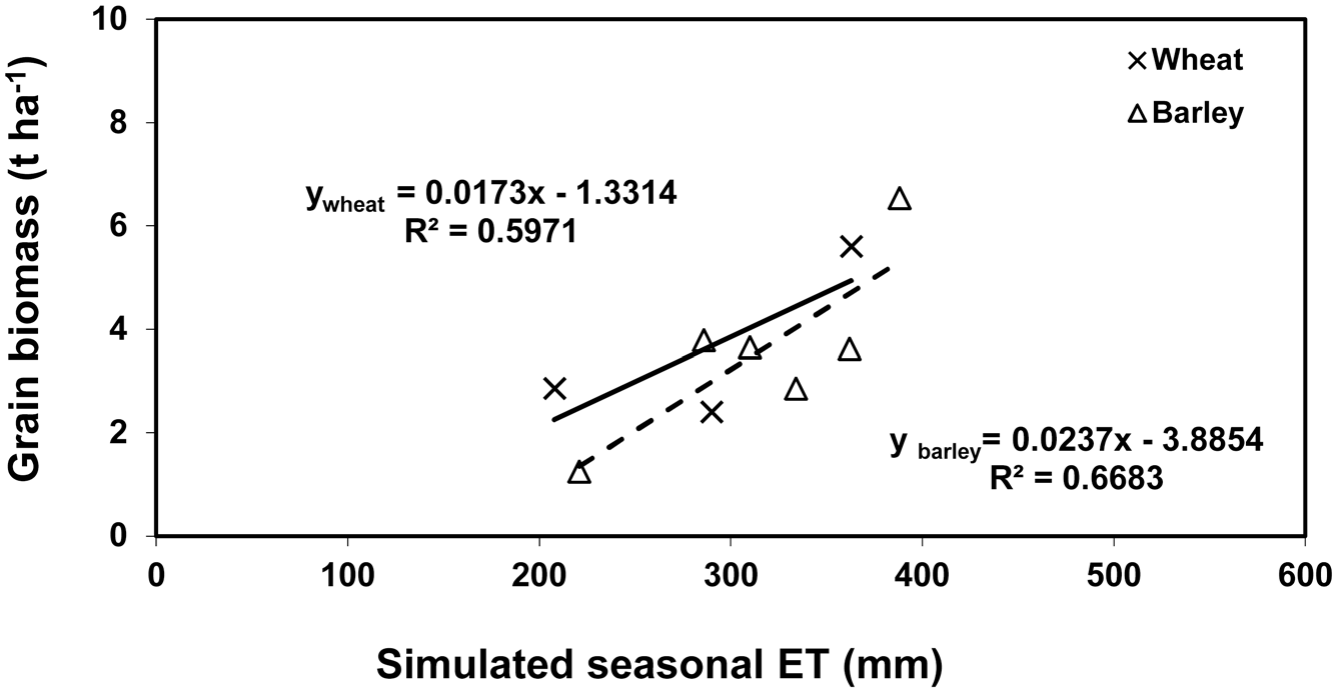
The relationships between the measured grain yield of barley and wheat and the simulated seasonal ET.

The effects of seasonal ET on grain yield of wheat and barley have long been investigated experimentally or by combined experimental and modelling approaches, and numerous studies have reported that grain yield is positively correlated with seasonal ET [23–29]. Many related the increase in grain yield with seasonal ET in a linear manner [23–24, 29]. The simulated results from 6 barley experiments and 3 wheat experiments in this study support the above findings. It indicates that grain yield is linearly related to seasonal ET ranging from 208 to 388 mm for both wheat and barley (Fig 8). However, it should be pointed out that such a relationship cannot be held for the whole possible ET range due to the plateau at the maximum yield. Regression analysis indicated that grain yield increased 17.3 and 23.7 kg ha^-l^ for every mm of water evapotranspired, in good agreement with the previous studies by Sharrat [23] and Zhang and Oweis [24]. Sharrat [23] reported a grain yield increase of 26 kg ha^-l^ for every mm of water evapotranspired over a range of 180 to 260 mm in seasonal ET for barley, whilst Zhang and Oweis [24] found that the corresponding figure for rainfed and irrigated bread wheat was 16 kg ha^-l^ for every mm of water evapotranspired over a range of 200 to 600 mm in seasonal ET. The positive relationships found in this study and in other previous studies suggest that in arid and semi-arid regions reservation of soil water and reduction of soil evaporation are critically important to increase grain yield. Options such as straw mulch and plastic film cover could be employed to reduce soil evaporation. Any reduction in soil evaporation could potentially save water for crop transpiration, and thus increase yield. Where possible, advanced precision irrigation systems using soil sensors and models should also be applied since the systems as such are increasingly becoming affordable and intelligent [55].

### Application of the model for agricultural water management

Optimizing agricultural water use is of great importance since agriculture is the biggest water consumer in the world and uses 70% world accessible fresh water [56]. It is reported that 40% of the world’s food is produced by irrigated agriculture with 60% of irrigated water wasted [56]. Nowadays irrigation is often applied based on experience instead of science. Excessive application of irrigated water not only results in considerable waste of water, but also causes negative physiological, environmental and ecological consequences [57–59]. It is well accepted that there exists a threshold of soil water content in the root zone below which crop transpiration and growth are reduced [55, 60]. The threshold soil water content, which varies with crop species, growth stages and soil texture, has been specified for a wide range of crops [20]. The importance of using agro-hydrological models for irrigation scheduling has been highlighted in recent review articles by Bastiaanssen et al. [1] and by Greenwood et al. [55]. The model used in this study could simulate root growth and soil water content in the entire root zone, and thus on any given day the averaged soil water content in the root zone could be calculated to compare the threshold for irrigation scheduling. The model as such has a potential to be applied not only for studying soil-crop water relations, but also for optimizing water use in crop production.

### Strategies to further improve model performance

Calibrated agro-hydrological models such as that employed in this study could be of great use for optimizing agricultural resources use, for minimizing the environmental impact, and for predicting crops response to weather. However, the calibration of agro-hydrological models is not always straightforward, and often leads to difficulties. The major sources causing the discrepancies between measurement and simulation are from the uncertainty of the model parameters involved in the complex system. The soil hydraulic properties are amongst those which are difficult to determine at the field scale as mentioned the above. A large body of literature is available on how to infer the effective soil hydraulic properties from laboratory experiments [50], field crop experiments [48] and field evaporation experiments [51–52].

Although there have been studies applying inverse modelling to infer the rooting depth [61–62], this approach has seldom been applied to root distribution parameters. Potentially inverse modelling could be used to deduce root parameters other than the rooting depth with equal success. In order for agro-hydrological models to perform satisfactorily, detailed description of the root system including individual roots is not necessary, but the information of root length density distribution in the soil profile is essential. Also, the utilization of water stress compensated models, i.e root water stress in one part of the root zone can be compensated for by enhanced extraction from the other wetter parts [63–65], could further enhance model performance. Although there are various equations proposed for considering the water stress compensation, the adoption of the equation and the determination of parameter values should be carefully selected based on the crop species.

Efforts should therefore be made to identify the parameters describing root length distribution and water stress compensation using the soil water measurements from different depths in the profile. Such approaches are now increasingly becoming possible as soil sensors become more accurate, affordable and more widely adopted [55]. Numerous optimization algorithms, traditional or evolutional, are readily available for the purpose of inverse modelling [51, 66–68]. Since parameter uniqueness and identifiability increases with a reduction in the number of parameters [67], the number of parameters to be identified should be kept to minimum. A two-staged inverse modelling strategy could be devised to maximize model performance: 1) to use the soil water measurements gathered from fallow soils for the identification of soil hydraulic properties; and 2) to use the data collected from the soil covered by crops together with the already identified soil hydraulic properties to identify parameters describing root dynamics such as root length distribution and root depth and root water stress compensation.

## Conclusions

The simulations of water dynamics for the soil-winter wheat/barley were carried out over a 10-year experimental period. The model satisfactorily reproduced the soil water measurements at various depths in the profile during growing seasons. The good agreement between measurement and simulation suggests: 1) the model could be reliably applied to modelling water dynamics in the soil-crop system; 2) the soil hydraulic properties estimated using the PTFs approach are generally representative for the soil studied; 3) the model simulated a linear relationship between wheat/barley grain yield and the simulated seasonal ET. The increases in grain yield were 17.3 and 23.7 kg ha^-l^ for every mm of water evapotranspired over the range of 180 to 288 mm in seasonal ET for wheat and barley, respectively. Future work should focus on improving model parameter estimates including those describing soil hydraulic properties and root development dynamics using a two-staged inverse modelling technique.

## Supporting Information

S1 Table Weather data measured during the experimental period from 2000–2010. (XLS)

S2 Table Measured soil water content at the depths of 15 cm, 45 cm and 75 cm during the experiment from 2006 to 2007. (XLS)

S3 Table Measured soil water content at the depths of 15 cm and 75 cm during the experiment from 2009 to 2010. (XLS)
